# Atrial Fibrillation and Clinical Outcomes in a Cohort of Hospitalized Patients with Sars-Cov-2 Infection and Chronic Kidney Disease

**DOI:** 10.3390/jcm10184108

**Published:** 2021-09-11

**Authors:** Simonetta Genovesi, Paola Rebora, Giuseppe Occhino, Emanuela Rossi, Alessandro Maloberti, Michele Belli, Paolo Bonfanti, Cristina Giannattasio, Claudio Rossetti, Oscar Massimiliano Epis, Nicola Ughi, Maria Grazia Valsecchi

**Affiliations:** 1 School of Medicine and Surgery, Milano-Bicocca University, 20126 Milan, Italy; alessandro.maloberti@unimib.it (A.M.); m.belli8@campus.unimib.it (M.B.); paolo.bonfanti@unimib.it (P.B.); 2 Cardiology Unit, Istituto Auxologico Italiano, IRCCS, 20100 Milan, Italy; 3 Bicocca Bioinformatics Biostatistics and Bioimaging Centre—B4, School of Medicine and Surgery, Milano-Bicocca University, 20126 Milan, Italy; paola.rebora@unimib.it (P.R.); g.occhino@campus.unimib.it (G.O.); emanuela.rossi@unimib.it (E.R.); grazia.valsecchi@unimib.it (M.G.V.); 4 Cardiology 4, ASST GOM Niguarda Hospital, 20162 Milan, Italy; cristina.giannattasio@unimib.it; 5 Department of Infectious Diseases, San Gerardo Hospital, 20900 Monza, Italy; 6 Nuclear Medicine, ASST GOM Niguarda Ca’ Granda, 20162 Milan, Italy; claudio.rossetti@ospedaleniguarda.it; 7 Division of Rheumatology, Multispecialist Medical Department, ASTT GOM Niguarda Ca’ Granda, 20162 Milan, Italy; oscar.epis@ospedaleniguarda.it (O.M.E.); nicola.ughi@ospedaleniguarda.it (N.U.)

**Keywords:** COVID-19, chronic kidney disease, atrial fibrillation, mortality, acute kidney injury

## Abstract

The aim of the study was to investigate the role of chronic kidney disease (CKD) on in-hospital mortality and on incident atrial fibrillation (AF) in patients infected with SARS-CoV-2. The incidence of acute kidney injury (AKI) was also investigated. Multivariable regression models were used to assess the association between renal function groups (estimated Glomerular Filtration Rate, eGFR, >60 mL/min, 30–59 mL/min, <30 mL/min) and in-hospital all-cause mortality and incident AF and AKI. A cohort of 2816 patients admitted in one year for COVID-19 disease in two large hospitals was analyzed. The independent predictors of mortality were severe CKD [HR 1.732 (95%CI 1.264–2.373)], older age [HR 1.054 (95%CI 1.044–1.065)], cerebrovascular disease [HR 1.335 (95%CI (1.016–1.754)], lower platelet count [HR 0.997 (95%CI 0.996–0.999)], higher C-reactive protein [HR 1.047 (95%CI 1.035–1.058)], and higher plasma potassium value 1.374 (95%CI 1.139–1.658). When incident AKI was added to the final survival model, it was associated with higher mortality [HR 2.202 (1.728–2.807)]. Incident AF was more frequent in patients with CKD, but in the multivariable model only older age was significantly related with a higher incidence of AF [OR 1.036 (95%CI 1.022–1.050)]. Incident AF was strongly associated with the onset of AKI [HR 2.619 (95%CI 1.711–4.009)]. In this large population of COVID-19 patients, the presence of severe CKD was an independent predictor of in-hospital mortality. In addition, patients who underwent AKI during hospitalization had a doubled risk of death. Incident AF became more frequent as eGFR decreased and it was significantly associated with the onset of AKI.

## 1. Introduction

Since COVID-19, caused by the severe acute respiratory syndrome coronavirus 2 (SARS-CoV-2), was discovered and evolved into a pandemic, it has become a source of interest for numerous studies [1,2]. Initially, the SARS-CoV-2 infection was considered to compromise lung function only, but subsequently, several studies showed that many other organs could be involved, the kidneys in particular [3,4]. The main symptoms of SARS-CoV-2 infection are observed 2–14 days after exposure. Symptoms include fever, cough, and difficulty breathing [5]. A severe complication of the disease is progressive respiratory failure, and death may occur in 3.4% of the infected patients [6]. As more studies are becoming available, the knowledge of those categories of patients at increased risk of hospitalization and poor outcomes is now improving. Chronic kidney disease (CKD) has been shown to be a comorbidity associated with reduced survival in patients hospitalized for COVID-19 disease [7,8].

Few data are available about COVID-19 disease and arrhythmias, in particular atrial fibrillation (AF) [9], and even less is known about this phenomenon in CKD patients [10].

As such, the aim of the study was to investigate the role of CKD on in-hospital mortality and on incident AF in patients infected with SARS-CoV-2. The incidence of acute kidney injury (AKI) was also assessed.

## 2. Materials and Methods

### 2.1. Study Design

This multicenter study included two large hospitals in Northern Italy, the San Gerardo Hospital in Monza and the Niguarda Hospital in Milan. Adult (≥18 years of age) patients diagnosed with COVID-19 and admitted in the two centers from 27 February 2020 to 3 January 2021 were included in the study. Clinical data were merged with the hospital lab data-base and patients with at least one creatinine test taken within three days from admission were included in the study. Participants were followed-up until the first occurrence of either hospital discharge, transfer to another facility, or death.

The study (STORM) was approved by the National Institutional Review Board (Spallanzani Hospital), ClinicalTrials.gov: NCT04424992.

### 2.2. Renal Function Estimation

Serum creatinine measurements were used to calculate the estimated Glomerular Filtration Rate (eGFR) by using the Chronic Kidney Disease Epidemiology Collaboration (CKD-EPI) equation [11]. Chronic kidney disease was defined as eGFR < 60 mL/min. Patients with eGFR between 30 and 59 mL/min were considered to have moderate CKD and those with eGFR < 30 mL/min to have severe CKD.

### 2.3. Definition of Covariates

Study covariates included age, sex, history of comorbidities, and blood chemistry parameters. The comorbidities that were taken into consideration were ischemic heart disease, congestive heart failure, peripheral vascular disease, cerebrovascular disease, chronic pulmonary disease, and diabetes. The history of AF and the percentage of permanent AF were included among the comorbidities as well. Blood chemistry parameters included, apart from creatinine, white blood cell (WBC) count, hemoglobin, hematocrit, platelets count, lymphocytes, C-reactive protein (CRP), urea, sodium, and potassium.

### 2.4. End-Points

The primary end-points were all-cause mortality and incident AF, defined as the first appearance of an AF episode during a hospital stay. The secondary end-point was AKI, defined as an increase in serum creatinine (SCr) by ≥0.3 mg/dL observed within 48 h; or an increase in SCr to ≥1.5 times the baseline creatinine value [12].

### 2.5. Statistical Analysis

The study population was subdivided into three groups: normal renal function, moderate CKD, and severe CKD, according to eGFR (>60, 30–59, and <30 mL/min, respectively). Continuous data were described by medians and quartiles (first-third Q1–Q3) and compared using the Kruskal–Wallis test by ranks, while categorical data were described by counts and percentages and compared by the Chi-square (χ^2^) test. Chronic kidney disease group was defined at each SCr measurement, and CKD stage at admission and at the last measurement before discharge was defined in subjects with at least two creatinine measurements.

The Aalen–Johansen estimator was used to estimate the crude cumulative incidence of mortality accounting for discharge as competing event, and the Gray test was used to test the null hypothesis of no difference in mortality among the three groups. A cause-specific Cox proportional-hazards regression model was used for investigating the association between CKD groups and all-cause mortality. Logistic regressions were applied to evaluate the association of CKD with the occurrence of AF and AKI during hospitalization. Patients with permanent AF were excluded from the model on AF, while patients with only one SCr measurement were excluded from the AKI model.

Potential confounders considered in the models were age, sex, ischemic heart disease, congestive heart failure, peripheral vascular disease, cerebrovascular disease, chronic pulmonary disease, diabetes, platelet count, and plasma concentration of hemoglobin, C-reactive protein and potassium. Odds ratios (ORs) or Hazard Ratios (HRs) with 95% confidence intervals (CIs) were reported. SAS 9.4 was used for the statistical analyses and the first type error was set at 0.05 (two-tailed).

## 3. Results

A total of 3308 patients, admitted for COVID-19 disease from February 2020 to January 2021, were recruited. Four hundred ninety-two subjects were excluded (30 with less than 18 years of age and 462 with no baseline measurement of creatinine) leaving a total of 2816 analyzed. The main clinical characteristics at the time of hospital admission and blood chemistry parameters at baseline are described in Table 1: 1981 (70%) patients had an eGFR > 60 mL/min, 636 (23%) had moderate CKD, and 199 (7%) had severe CKD. Chronic kidney disease patients were older than those with eGFR > 60 mL/min and the number of comorbidities increased as renal function decreased. Patients with CKD had lower values of hemoglobin, hematocrit, and platelet count and higher inflammatory indices and plasma potassium values compared to those with preserved renal function. 

### 3.1. Mortality

**Figure 1:**
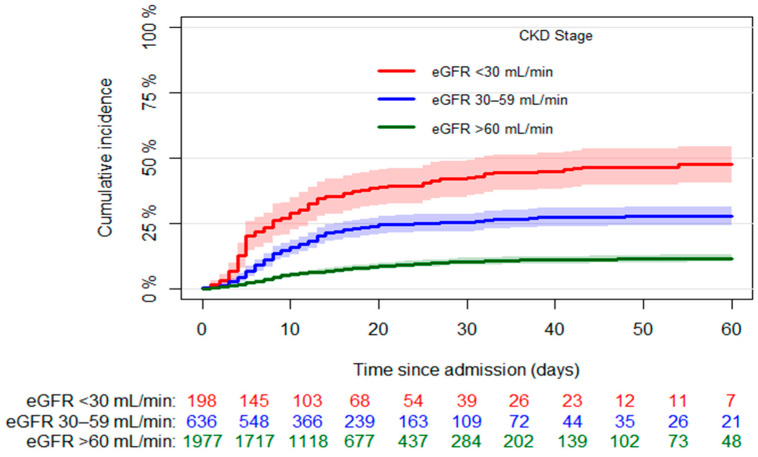
Crude in-hospital mortality stratified by CKD, with number of patients at risk in three groups. Abbreviations: CKD, chronic kidney disease; eGFR, estimated glomerular filtration rate.

The median hospital stay was 13 days (Q1–Q3 8–22) and 504 cases of death (17.9%) were observed, with 233 (11.8%) in the group of patients with eGFR > 60 mL/min, 177 (27.8%) in that of patients with eGFR 30–59 mL/min, and 94 (47.5%) in that of patients with eGFR < 30 mL/min (*p* < 0.001). Figure 1 shows the mortality incidence observed in the three groups over the first 60 days of hospitalization.

In the model, adjusted for CKD stage, age, sex and comorbidities, including incident AF, factors significantly associated with higher mortality were the presence of CKD [HR 1.307 (95%CI 1.056–1.617) and HR 2.146 (95%CI 1.649–2.794) for eGFR 30–59 mL/min and <30 mL/min, respectively], older age [HR 1.049 (95%CI 1.041–1.058)], and male sex [HR 1.557 (95%CI 1.277–1.900). Ischemic heart disease and cerebrovascular disease were slightly associated with lower survival (Table A1).

In the model, adjusted for CKD stage, age, and sex, which included laboratory tests, the variables significantly associated with increased mortality were severe CKD [HR 1.621 (95%CI 1.166–2.253)], older age [HR 1. 057 (95%CI 1.046–1.067)], lower platelet count [HR 0.997 (95%CI 0.996–0.999)], and higher C-reactive protein [HR 1.045 (95%CI 1.034–1.056)] and plasma potassium [HR 1.371 (95%CI 1.135–1.657)] levels (Table A2).

The final model showed that the independent predictors of mortality were severe CKD [HR 1.732 (95%CI 1.264–2.373)], older age [HR 1.054 (95%CI 1.044–1.065)], cerebrovascular disease [HR 1.335 (95%CI 1.016–1.754)], lower platelet count [HR 0.997(95%CI 0.996–0.999)], and higher C-reactive protein [HR 1.047 (95%CI 1.035–1.058)] and plasma potassium values [HR 1.374 (95%CI 1.139–1.658)] (Table 2).

Two hundred and ten out of 482 (43.6%) patients who suffered from AKI died. Among the AKI patients, 247 had an eGFR > 60 mL/min at baseline, and 88 (35.6%) of those underwent in-hospital mortality, whereas among patients with AKI and eGFR < 60 mL/min, deaths were 122 out of 235 (51.9%) (*p* = 0.0003). When incident AF and incident AKI were added to the final survival model, AKI was found to be strongly associated with higher mortality [HR 2.202 (1.728–2.807)], whereas CKD was no longer so. There was no association between incident AF and survival (Table 3).

### 3.2. Atrial Fibrillation

**Figure 2:**
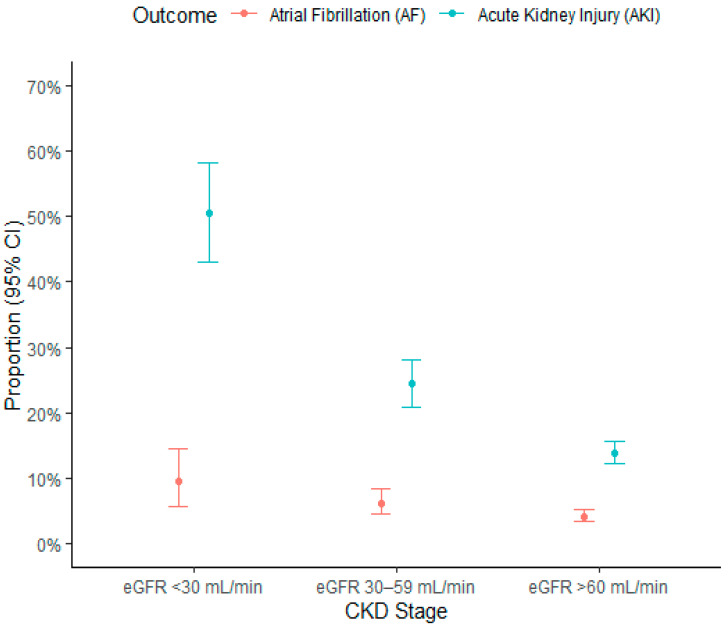
Proportion plot with 95% confidence interval for AF (in 2720 patients without permanent AF) and AKI (in 2550 patients with at least two creatinine measurements) according to chronic kidney disease. Abbreviations: AF, atrial fibrillation; AKI, acute kidney injury; CKD, chronic kidney disease; eGFR, estimated glomerular filtration rate.

Six percent of patients (*n* = 170) had a history of AF; the arrhythmia was permanent in 3.4% of cases. The characteristics of patients with permanent AF and of the rest of the population are shown in Table A3. The presence of a history of AF was found to be more frequent in patients with worse renal function: 10.2% in those with eGFR between 30 and 59 mL/min and 16.6% in those with eGFR < 30 mL/min against 3.6% in individuals with eGFR > 60 mL/min. During hospitalization, 143 (5.1%) patients suffered an incident episode of AF. Incident AF was more frequent in patients with CKD and the rate increased as the stage of CKD worsened: 84 (4.2%) in the subgroup of patients with eGFR > 60 mL/min, 40 (6.3%) in that of patients with eGFR 30–59 mL/min, and 19 (9.5%) in that of patients with eGFR < 30 mL/min (*p* = 0.001, Figure 2).

Other arrhythmias (ventricular arrhythmias or brady-arrhythmias) were recorded in 30 (1.1%) subjects and their incidence did not seem to be influenced by renal function. Table 4 shows the logistic regression model on AF incidence by CKD adjusted by age and sex. Only older age was significantly associated with a higher incidence of AF [OR 1.036 (95%CI 1.022–1.050)].

The addition of other possible confounding factors to the model did not change the result (Table A4, Model A). When age was removed from the statistical models, the presence of CKD was the only factor significantly associated with incident AF (Table A4, Model B and Model C).

### 3.3. Acute Kidney Injury

Data on AKI were available in 2550 patients. The characteristics of patients with a single creatinine measurement and of the rest of the population are shown in Table A5. During hospitalization, 482 (18.9%) patients experienced AKI. This event occurred more frequently in patients with CKD: 248 out of 1784 (13.9%) in the subgroup of patients with eGFR > 60 mL/min, 143 out of 586 (24.4%) in patients with eGFR 30–59 mL/min, and 91 out of 180 (50.6%) in those with eGFR < 30 mL/min (*p* < 0.001, Figure 2).

With multivariable logistic regression, the factors that were significantly associated with the occurrence of AKI were CKD [HR 1.782 (95%CI 1.334–2.382) and HR 4.142 (95%CI 2.760–6.216) for eGFR 30–59 mL/min and <30 mL/min compared to eGFR >60 mL/min, respectively], male sex [HR 1. 434 (95%CI 1.097–1.875)], the presence of peripheral vascular disease [HR 1.791 (95%CI 1.212–2.646)], lower hemoglobin values [HR 0.899 (95%CI 0.844–0.958)], and higher C-reactive protein values [HR 1.048 (95%CI 1.034–1.062)]. Incident AF was strongly associated with the onset of AKI [HR 2.619 (95%CI 1.711–4.009)], even after adjustment for possible confounding factors (Table 5). When a cause-specific hazard regression model for AKI was fitted, the results were superimposable to those of the logistic model (Table A6).

### 3.4. Renal Function

Table 6 shows the distribution of the population according to CKD status at hospital admission and at the last evaluation before discharge of 2099 subjects discharged alive and with at least two creatinine values measurements.

Overall, 73 out of 2099 (3.5%) patients experienced a worsening of their renal function: 64 with eGFR > 60 mL/min at hospital admission entered the moderate or severe CKD stage and 9 patients with moderate CKD worsened towards severe CKD. Among patients with deterioration of renal function, 4 out of 73 (5.5%) experienced an episode of incident AF, while the event occurred in 82 out of 2026 (4.1%) patients without renal function decline (*p* = 0.5395). Thirty four out of 73 (46.6%) patients with worsening renal function had AKI compared to 237 out of 2026 (11.7%) in patients who maintained their renal function unchanged during their hospital stay (*p* < 0.0001). It is interesting to note that on the other hand, 248 out of 2099 (11.8%) patients, experienced an improvement of their renal function had at discharge: 207 with eGFR 30–59 mL/min and 41 with eGFR < 30 mL/min at baseline.

## 4. Discussion

In a large population of patients hospitalized for COVID-19 disease, the presence of severe CKD on admission is an independent predictor of in-hospital mortality. The number of cases of incident AF increases as eGFR decreases, but renal function does not predict incident AF after correction for possible confounders. In contrast, incident AF is significantly associated with the onset of AKI.

Approximately one third of the study population had CKD at hospital admission, a finding in agreement with that described in other studies, that have reported a prevalence of CKD in COVID-19 patients between 22 and 38% [13,14]. The incidence of in-hospital mortality in this study was 18%. As already described by other authors [15,16,17], the presence of reduced renal function increases mortality rate. In most studies, CKD is defined as the presence of eGFR <60 mL/min, with no further differentiation between the various stages of renal disease. Importantly, in our study, a significant increase in mortality was evident as the stage of CKD worsened (from 28% in patients with moderate CKD to 48% in those with severe CKD). After adjustment for age and comorbidities, the presence of eGFR between 30 and 59 mL/min was significantly associated with a 30% increase in mortality, and that of eGFR < 30 mL/min doubled the risk of death. When factors related to the severity of COVID-19 disease were also included in the model, only severe CKD and not moderate CKD remained an independent predictor of reduced in-hospital survival (HR 1.74). When incident AKI was added to the final survival model, it was a predictor of mortality, whereas CKD was no longer so. This finding strongly suggests that AKI is a mediator of mortality in COVID-19 disease, particularly in patients with CKD at baseline: CKD patients who die experience an abrupt impairment of renal function before death. In addition, some patients who undergo AKI die even if they show normal eGFR at baseline. Previously, a meta-analysis including 26 studies (*n* = 5497 patients) showed that, in COVID-19 patients, the presence of AKI was associated with a more than 10-fold increased risk of mortality [18]. An interesting finding of our study is the strong association between higher potassium values and mortality. Various studies have described hyperkalemia as a risk factor for survival in populations with several types of heart or kidney diseases. [19,20,21]. The present study shows that this relationship is also confirmed in COVID-19 patients, independently of the presence of CKD.

Atrial fibrillation is a frequently occurring arrhythmia in patients with CKD [22,23,24] and an increased incidence has been described in critically ill patients [25]. The presence of pre-existing CKD is by itself a risk factor for AF in patients with acute sepsis [26]. Few authors have studied the association between AF and COVID-19 disease. In patients hospitalized in cardiology wards for Sars-CoV-2 infection, the Cardio-COVID-Italy study showed an incidence of in-hospital AF of 12.5% [27], while García-Granja et al. observed an incidence of 10.4% [9]. Russo et al. found that in patients with COVID-19 admitted to emergency department, the incidence of AF was higher, equal to 17.1% [10]. To our knowledge, there are very few data about the relationship between incident AF and renal function in COVID-19 patients. Russo et al. showed an association between CKD and incident AF in patients admitted in emergency units for COVID-19 disease, which, however, was not confirmed after adjustment for possible confounders [10]. As expected, CKD patients in our study population had a higher prevalence of a positive history of AF. Episodes of incident AF increased as eGFR decreased, approaching 10% in patients with eGFR < 30 mL/min. This phenomenon was closely associated with the higher age of patients with reduced renal function (median 78 vs. 59 years of age). However, it is interesting to point out that in our population, when age was removed from the confounding factors in the multivariable model, a significant association with a higher incidence of AF was found only for the presence of CKD and not for the cardiac comorbidities. It is likely that the factors that predisposed CKD patients to be more liable to AF were only indirectly associated with the presence of reduced eGFR (older age, higher prevalence of heart disease, frequent presence of structural remodeling and cardiac fibrosis) and that this is the reason why CKD, although associated with a higher incidence of AF, was not found to be an independent predictive factor. In our study population, incident AF did not have an impact on in-hospital mortality, however, it was closely associated with the onset of AKI, which in turn appears to be an important mediator of mortality. It will be interesting to see whether in-hospital AF might have an effect on patients’ long-term survival after discharge.

The incidence of AKI in our patients was 19%, in agreement with the incidence reported in previous studies that ranges from 18% to 30.6% [28,29]. Various studies have described AKI in COVID-19 patients and the factors associated with this outcome [30,31]. This phenomenon is probably underestimated in our population. In fact, we see that a percentage of patients (12%) improved their CKD stage at discharge, suggesting that these are individuals who arrived in the emergency department with an ongoing AKI that resolved as the disease-causing admission resolved. Understanding the true incidence of COVID-19-related AKI is important, as our data demonstrated that patients who experienced an AKI event left the hospital with worsened kidney function more often than hospitalized patients without AKI. Our results indicate lower hemoglobin levels and higher C-reactive protein values as factors that may be associated with in-hospital AKI, suggesting the importance of considering subjects with low hematocrit with special care, especially in case of elevated inflammatory indices. A new and interesting result of the present study is the association between AKI and incident AF. It has been previously described that the arrhythmia most frequently associated with AKI in the intensive care units is AF [25]. The onset of AF in critically ill patients is an independent predictor of AKI [32,33]; in turn, the incidence of AF is almost double in critically ill patients who suffered AKI compared to those with normal renal function [34]. In our population, the risk of AKI is two and a half times higher in patients with incident AF. One reason could be that the onset of arrhythmia creates hemodynamic instability, with a reduction in left ventricular ejection fraction and a consequent drop in renal perfusion. From our results, it seems that the subjects at greater risk of experiencing AKI are those with lower hemoglobin values and higher indices of inflammation. It is possible to hypothesize that the association of the three factors (anemia, inflammation, and arrhythmia) may constitute a critical clinical situation for the onset of AKI.

In conclusion, although the data were collected retrospectively, our study provides some new information that may be clinically useful in understanding the complex relationship between kidney function and Sars-CoV-2 infection. The mortality rate from COVID-19 is not only higher in CKD patients (with eGFR< 60 mL/min), but it also increases significantly when moving from moderate to severe CKD. In addition, patients who underwent AKI during hospitalization had a doubled risk of death. COVID-19 appears to increase the incidence of in-hospital AF, particularly in patients with CKD, and, in turn, patients who experience an episode of in-hospital AF are also more likely to suffer from AKI.

## Figures and Tables

**Table 1:** Baseline characteristics (overall and by renal function).

	Overall	eGFR < 30 mL/min	eGFR 30–59 mL/min	eGFR > 60 mL/min	*p*-Value	Missing (%)
n	2816	199	636	1981		
Male (%)	1856 (65.9)	126 (63.3)	391 (61.5)	1339 (67.6)	0.013	0
Age (median [IQR])	65 [54, 77]	78 [68, 86]	77 [68, 83]	59 [51, 70]	<0.001	0
eGFR (median [IQR])	77.13 [55.65, 93.09]	19.95 [13.02, 25.85]	47.61 [40.36, 54.59]	86.52 [75.17, 97.93]	-	0
History of AF (%)	170 (6.0)	33 (16.6)	65 (10.2)	72 (3.6)	<0.001	0
Permanent AF (%)	96 (3.4)	18 (9.0)	43 (6.8)	35 (1.8)	<0.001	0
Ischemic Heart Disease (%)	263 (9.3)	43 (21.6)	104 (16.4)	116 (5.9)	<0.001	0.1
Congestive Heart Failure (%)	120 (4.3)	35 (17.6)	51 (8.0)	34 (1.7)	<0.001	0.1
Peripheral Vascular Disease (%)	206 (7.3)	39 (19.6)	78 (12.3)	89 (4.5)	<0.001	0.1
Cerebrovascular Disease (%)	255 (9.1)	28 (14.1)	81 (12.8)	146 (7.4)	<0.001	0.1
Chronic Pulmonary Disease (%)	245 (8.7)	32 (16.1)	92 (14.5)	121 (6.1)	<0.001	0.1
Diabetes (%)	477 (17.0)	67 (33.7)	145 (22.8)	265 (13.4)	<0.001	0.1
White Blood Cells (103 U/L) (median [IQR])	6.76 [4.88, 9.98]	7.53 [5.12, 11.68]	7.18 [4.98, 10.48]	6.53 [4.82, 9.46]	0.004	3.5
Hemoglobin (g/dL) (median [IQR])	12.90 [11.55, 14.00]	11.10 [9.60, 12.40]	12.50 [11.10, 13.70]	13.10 [11.90, 14.20]	<0.001	3.3
Hematocrit (%) (median [IQR])	38.70 [35.10, 42.10]	33.85 [29.78, 38.35]	38.00 [34.20, 41.40]	39.30 [35.90, 42.40]	<0.001	3.3
Platelets (103 U/L) (median [IQR])	209.00 [157.00, 269.00]	188.00 [132.00, 243.25]	198.00 [145.00, 260.00]	215.00 [164.00, 277.00]	<0.001	3.3
Lymphocytes (103 U/L) (median [IQR])	1.00 [0.71, 1.40]	0.81 [0.59, 1.17]	0.91 [0.63, 1.32]	1.07 [0.75, 1.44]	<0.001	6.7
C-Reactive Protein (mg/dL) (median [IQR])	6.66 [2.60, 12.20]	9.30 [3.50, 15.18]	7.70 [3.20, 13.61]	6.34 [2.38, 11.46]	<0.001	12.1
Urea (mg/dL) (median [IQR])	39.00 [28.00, 59.00]	118.00 [95.00, 169.25]	61.00 [46.00, 80.00]	33.00 [25.00, 44.00]	<0.001	12.6
Creatinine (mg/dL) (median [IQR])	0.98 [0.80, 1.20]	2.69 [2.23, 4.00]	1.33 [1.19, 1.53]	0.89 [0.74, 1.00]	-	0
Sodium (mmol/L) (median [IQR])	139.00 [137.00, 141.00]	140.00 [137.00, 143.00]	139.00 [137.00, 142.00]	139.00 [137.00, 141.00]	0.01	8.2
Potassium (mmol/L) (median [IQR])	4.20 [3.84, 4.52]	4.50 [4.19, 5.00]	4.24 [3.88, 4.60]	4.11 [3.80, 4.47]	<0.001	9.2

Abbreviations: AF, atrial fibrillation, CKD, chronic kidney disease; eGFR, estimated glomerular filtration rate; *n*, number; IQR, inter quartile range.

**Table 2:** Cox proportional hazards model on the effect of CKD on in-hospital mortality adjusted by age, sex, and selected clinical and laboratory variables. *n* = 2223, *n* of deaths = 371.

	HR (95% CI)	*p*-Value
CKD Stage (eGFR 30–59 mL/min vs. eGFR > 60 mL/min)	1.198 (0.934–1.538)	0.1550
CKD Stage (eGFR < 30 mL/min vs. eGFR > 60 mL/min)	1.732 (1.264–2.373)	0.0006
Age (years)	1.054 (1.044–1.065)	<0.0001
Sex (M vs. F)	1.191 (0.946–1.499)	0.1368
Ischemic Heart Disease (Yes vs. No)	1.225 (0.929–1.614)	0.1498
Cerebrovascular Disease (Yes vs. No)	1.335 (1.016–1.754)	0.0382
Platelets (10^3^ U/L)	0.997 (0.996–0.999)	<0.0001
C-Reactive Protein (mg/dL)	1.047 (1.035–1.058)	<0.0001
Potassium (mmol/L)	1.374 (1.139–1.658)	0.0009

Abbreviations: CKD, chronic kidney disease; *n*, number; eGFR, estimated glomerular filtration rate; HR, hazard ratio.

**Table 3:** Cox proportional hazards model on the effect of CKD on in-hospital mortality adjusted by age, sex, and selected clinical and laboratory variables, incident Atrial Fibrillation, and AKI. *n* = 2006, *n* of death = 327.

	HR (95% CI)	*p*-Value
CKD Stage (eGFR 30–59 mL/min vs. eGFR > 60 mL/min)	1.100 (0.841–1.437)	0.4864
CKD Stage (eGFR < 30 mL/min vs. eGFR > 60 mL/min)	1.152 (0.802–1.656)	0.4436
Age (years)	1.056 (1.045–1.068)	<0.0001
Sex (M vs. F)	1.225 (0.955–1.571)	0.1108
Ischemic Heart Disease (Yes vs. No)	1.120 (0.826–1.518)	0.4656
Cerebrovascular Disease (Yes vs. No)	1.357 (1.004–1.835)	0.0472
Platelets (10^3^ U/L)	0.998 (0.997–0.999)	0.0028
C-Reactive Protein (mg/dL)	1.038 (1.026–1.051)	<0.0001
Potassium (mmol/L)	1.379 (1.129–1.685)	0.0017
Incident Atrial Fibrillation (Yes vs. No)	1.080 (0.757–1.543)	0.6705
AKI (Yes vs. No)	2.202 (1.728–2.807)	<0.0001

Abbreviations: CKD, chronic kidney disease; *n*, number; eGFR, estimated glomerular filtration rate; HR, hazard ratio; AKI, acute kidney injury.

**Table 4:** Logistic model on the CKD effect on incident AF adjusted by age and sex. Patients with permanent AF were excluded from the model (*n* = 96). *n* = 2720, AF = 143.

	OR (95% CI)	*p*-Value
CKD Stage (eGFR 30–59 mL/min vs. eGFR > 60 mL/min)	1.005 (0.656–1.540)	0.9806
CKD Stage (eGFR < 30 mL/min vs. eGFR > 60 mL/min)	1.547 (0.882–2.713)	0.1279
Age (years)	1.036 (1.022–1.050)	<0.0001
Sex (M vs. F)	1.319 (0.910–1.911)	0.1441

Abbreviations: AF, atrial fibrillation; CKD, chronic kidney disease; *n*, number; eGFR, estimated glomerular filtration rate; OR, odds ratio.

**Table 5:** Logistic model on the CKD effect on AKI during hospitalization adjusted by age, sex, comorbidities, incident AF, and relevant laboratory measurements. *n* = 2200, AKI = 399.

	OR (95% CI)	*p*-Value
CKD Stage (eGFR 30–59 mL/min vs. eGFR > 60 mL/min)	1.782 (1.334–2.382)	<0.0001
CKD Stage (eGFR < 30 mL/min vs. eGFR > 60 mL/min)	4.142 (2.760–6.216)	<0.0001
Age (years)	0.997 (0.988–1.007)	0.5934
Sex (M vs. F)	1.434 (1.097–1.875)	0.0083
Ischemic Heart Disease (Yes vs. No)	0.954 (0.640–1.423)	0.8184
Congestive Heart Failure (Yes vs. No)	1.401 (0.829–2.366)	0.2077
Peripheral Vascular Disease (Yes vs. No)	1.791 (1.212–2.646)	0.0035
Cerebrovascular Disease (Yes vs. No)	0.763 (0.494–1.176)	0.2202
Chronic Pulmonary Disease (Yes vs. No)	0.740 (0.490–1.118)	0.1524
Diabetes (Yes vs. No)	1.171 (0.872–1.572)	0.2939
Hemoglobin (g/dL)	0.899 (0.844–0.958)	0.0010
C-Reactive Protein (mg/dL)	1.048 (1.034–1.062)	<0.0001
Incident Atrial Fibrillation (Yes vs. No)	2.619 (1.711–4.009)	<0.0001

Abbreviations: AF, atrial fibrillation; AKI, acute kidney injury; CKD, chronic kidney disease; eGFR, estimated glomerular filtration rate; *n*, number; OR, odds ratio.

**Table 6:** Distribution of the population according to CKD at hospital admission and at the last evaluation before discharge.

CKD at Admission *n* (%)	CKD at Discharge *n* (%)			
	eGFR < 30 mL/min	eGFR 30–59 mL/min	eGFR > 60 mL/min	Total (%)
eGFR < 30 mL/min	58 (58.6%)	35 (35.4%)	6 (6.1%)	99 (100%)
eGFR 30–59 mL/min	9 (2.1%)	219 (50.3%)	207 (47.6%)	435 (100%)
eGFR > 60 mL/min	4 (0.3%)	60 (3.4%)	1501 (95.9%)	1565 (100%)
Total	71	314	1714	2099

Abbreviations: CKD, chronic kidney disease; eGFR, estimated glomerular filtration rate; *n*, number.

## Data Availability

Data are available on reasonable request from the corresponding author.

## COVID-STORM Clinicians

Paolo Bonfanti, Andrea Biondi, Giuseppe Foti, Giacomo Bellani, Giuseppe Citerio, Ernesto Contro, Alberto Pesci, Maria Grazia Valsecchi, Marina Cazzaniga.

## COVID-STORM Data Management

Davide Gaudesi, Emanuela Rossi, Nicoletta Cordani.

## Appendix A

**Table A1:** Cox proportional hazards model on the impact of clinical factors on in-hospital mortality. Confounding variables with a significance level of <0.1 were retained in the final model. *n* = 2811, death = 504.

	HR (95% CI)	*p*-Value
CKD Stage (eGFR 30–59 mL/min vs. eGFR > 60 mL/min)	1.307 (1.056–1.617)	0.0138
CKD Stage (eGFR < 30 mL/min vs. eGFR > 60 mL/min)	2.146 (1.649–2.794)	<0.0001
Age (years)	1.049 (1.041–1.058)	<0.0001
Sex (M vs. F)	1.557 (1.277–1.900)	<0.0001
Ischemic Heart Disease (Yes vs. No)	1.250 (0.983–1.590)	0.0687
Congestive Heart Failure (Yes vs. No)	1.154 (0.833–1.598)	0.3899
Peripheral Vascular Disease (Yes vs. No)	0.972 (0.742–1.273)	0.8349
Cerebrovascular Disease (Yes vs. No)	1.261 (0.990–1.606)	0.0607
Chronic Pulmonary Disease (Yes vs. No)	0.916 (0.699–1.202)	0.5281
Diabetes (Yes vs. No)	0.985 (0.799–1.215)	0.8893
Atrial Fibrillation (Yes vs. No)	1.056 (0.796–1.400)	0.7071

Abbreviations: AF, atrial fibrillation; CKD, chronic kidney disease; *n*, numbers; eGFR, estimated glomerular filtration rate; HR, hazard ratio.

**Table A2:** Cox proportional hazards model on the impact of laboratories measurements on in-hospital mortality. Confounding variables with a significance level of <0.1 were retained in the final model. *n* = 2217, death = 369.

	HR (95% CI)	*p*-Value
CKD Stage (eGFR 30–59 mL/min vs. eGFR > 60 mL/min)	1.162 (0.903–1.495)	0.2420
CKD Stage (eGFR < 30 mL/min vs. eGFR > 60 mL/min)	1.621 (1.166–2.253)	0.0041
Age (years)	1.057 (1.046–1.067)	<0.0001
Sex (M vs. F)	1.239 (0.978–1.568)	0.0753
Hemoglobin (g/dL)	0.978 (0.926–1.033)	0.4297
Platelets (10^3^ U/L)	0.997 (0.996–0.999)	<0.0001
C-Reactive Protein (mg/dL)	1.045 (1.034–1.056)	<0.0001
Sodium (mmol/L)	1.019 (0.996–1.042)	0.1138
Potassium (mmol/L)	1.371 (1.135–1.657)	0.0011

Abbreviations: CKD, chronic kidney disease; *n*, numbers; eGFR, estimated glomerular filtration rate; HR, hazard ratio.

**Table A3:** Differences between patients with permanent AF and the rest of the population.

	**Permanent AF**	
	**No**	**Yes**
*n*	2720	96
CKD stage (%)		
eGFR < 30 mL/min	181 (6.7)	18 (18.8)
eGFR 30–59 mL/min	593 (21.8)	43 (44.8)
eGFR > 60 mL/min	1946 (71.5)	35 (36.5)
Sex = M (%)	1801 (66.3)	54 (56.2)
Age (median [range])	64 [18, 99]	80 [56, 99]
Ischemic Heart Disease = Yes (%)	240 (8.8)	23 (24.0)
Congestive Heart Failure = Yes (%)	101 (3.7)	19 (19.8)
Diabetes = Yes (%)	456 (16.8)	21 (21.9)
Hemoglobin (g/dL) (median [range])	12.90 [3.40, 17.90]	11.85 [6.50, 16.20]
Potassium (mmol/L) (median [range])	4.20 [2.60, 7.67]	4.20 [2.60, 6.00]

Abbreviations: AF, atrial fibrillation; *n*, number; CKD, chronic kidney disease; *n*, numbers; eGFR, estimated glomerular filtration rate; HR, hazard ratio.

**Table A4:** Logistic model on the impact of CKD on incident AF adjusted by age, sex, comorbidities and laboratory measurements (Model A), by sex (Model B) and by sex, comorbidities, and laboratory measurements (Model C) *n* = 2386, AF = 112.

	OR (95% CI)	*p*-Value
**Model A**		
CKD Stage (eGFR 30–59 mL/min vs. eGFR > 60 mL/min)	0.808 (0.496–1.318)	0.3935
CKD Stage (eGFR < 30 mL/min vs. eGFR > 60 mL/min)	1.210 (0.613–2.389)	0.5832
Age (years)	1.052 (1.034–1.069)	<0.0001
Sex (M vs. F)	1.504 (0.970–2.334)	0.0683
Ischemic Heart Disease (Yes vs. No)	0.738 (0.386–1.412)	0.3593
Congestive Heart Failure (Yes vs. No)	1.108 (0.475–2.585)	0.8127
Diabetes (Yes vs. No)	0.969 (0.595–1.577)	0.8984
Hemoglobin (g/dL)	0.980 (0.882–1.088)	0.7021
Potassium (mmol/L)	1.120 (0.798–1.571)	0.5124
**Model B**		
CKD Stage (eGFR 30–59 mL/min vs. eGFR > 60 mL/min)	1.620 (1.098–2.391)	0.0150
CKD Stage (eGFR < 30 mL/min vs. eGFR > 60 mL/min)	2.613 (1.548–4.410)	0.0003
Sex (M vs. F)	0.823 (0.823–1.712)	0.3582
**Model C**		
CKD Stage (eGFR 30–59 mL/min vs. eGFR > 60 mL/min)	1.509 (0.959–1.318)	0.0754
CKD Stage (eGFR < 30 mL/min vs. eGFR > 60 mL/min)	2.227 (1.159–4.280)	0.0163
Sex (M vs. F)	1.317 (0.853–2.035)	0.2143
Ischemic Heart Disease (Yes vs. No)	0.921(0.480–1.767)	0.8045
Congestive Heart Failure (Yes vs. No)	1.267 (0.546- 2.940)	0.5823
Diabetes (Yes vs. No)	1.096 (0.673–1.783)	0.7125
Hemoglobin (g/dL)	0.957 (0.863–1.062)	0.4105
Potassium (mmol/L)	1.062 (0.754–1.496)	0.7323

Abbreviations: AF, atrial fibrillation; CKD, chronic kidney disease; *n*, number; eGFR, estimated glomerular filtration rate; OR, odds ratio.

**Table A5:** Differences between patients with a single creatinine measurement and the rest of the population.

	Two sCr Measurements	
	No	Yes
*n*	267	2549
CKD stage (%)		
eGFR < 30 mL/min	19 (7.1)	180 (7.1)
eGFR 30–59 mL/min	50 (18.7)	586 (23.0)
eGFR > 60 mL/min	198 (74.2)	1783 (69.9)
Sex = M (%)	158 (59.2)	1697 (66.6)
Age (median [range])	60 [18, 99]	65 [18, 99]
Ischemic Heart Disease = Yes (%)	24 (9.0)	239 (9.4)
Congestive Heart Failure = Yes (%)	12 (4.5)	108 (4.2)
Peripheral Vascular Disease = Yes (%)	12 (4.5)	194 (7.6)
Cerebrovascular Disease = Yes (%)	33 (12.4)	222 (8.7)
Chronic Pulmonary Disease = Yes (%)	16 (6.0)	229 (9.0)
Diabetes = Yes (%)	36 (13.5)	441 (17.3)
Hemoglobin (g/dL) (median [range])	13.00 [6.50, 17.00]	12.80 [3.40, 17.90]
C-Reactive Protein (mg/dL) (median [range])	4.00 [0.00, 41.00]	7.09 [0.00, 47.80]
Incident Atrial Fibrillation = Yes (%)	9 (3.4)	134 (5.3)

Abbreviations: sCr, serum creatinine; AF, atrial fibrillation; *n*, number; CKD, chronic kidney disease; *n*, numbers; eGFR, estimated glomerular filtration rate.

**Table A6:** Cause-specific hazard regression model for AKI.

	HR (95% CI)	*p*-Value
CKD Stage (eGFR 30–59 mL/min vs. eGFR > 60 mL/min)	1.790 (1.396–2.296)	<0.0001
CKD Stage (eGFR < 30 mL/min vs. eGFR > 60 mL/min)	3.607 (2.641–4.928)	<0.0001
Age (years)	0.996 (0.989–1.004)	0.3599
Sex (M vs. F)	1.314 (1.044–1.654)	0.0198
Ischemic Heart Disease (Yes vs. No)	1.000 (0.723–1.385)	0.9979
Congestive Heart Failure (Yes vs. No)	1.156 (0.767–1.744)	0.4887
Peripheral Vascular Disease (Yes vs. No)	1.605 (1.180–2.183)	0.0026
Cerebrovascular Disease (Yes vs. No)	0.844 (0.582–1.225)	0.3731
Chronic Pulmonary Disease (Yes vs. No)	0.774 (0.545–1.099)	0.1523
Diabetes (Yes vs. No)	1.039 (0.814–1.327)	0.7566
Hemoglobin (g/dL)	0.939 (0.891–0.990)	0.0188
C-Reactive Protein (mg/dL)	1.034 (1.023–1.045)	<0.0001
Incident Atrial Fibrillation (Yes vs. No)	1.626 (1.181–2.238)	0.0029

Abbreviation: AKI, Acute Kidney Injury; CKD, chronic kidney disease; *n*, numbers; eGFR, estimated glomerular filtration rate; HR, hazard ratio.

## References

[1] Pascarella G., Strumia A., Piliego C., Bruno F., Del Buono R., Costa F., Scarlata S., Agrò F.E. (2020). COVID-19 diagnosis and management: A comprehensive review. J. Intern. Med..

[2] Wiersinga W.J., Rhodes A., Cheng A.C., Peacock S.J., Prescott H.C. (2020). Pathophysiology, Transmission, Diagnosis, and Treatment of Coronavirus Disease 2019 (COVID-19): A Review. JAMA.

[3] Nadim M.K., Forni L.G., Mehta R.L., Connor M.J.j., Liu K.D., Ostermann M., Rimmelé T., Zarbock A., Bell S., Bihorac A. (2020). COVID-19-associated acute kidney injury: Consensus report of the 25th Acute Disease Quality Initiative (ADQI) Workgroup. Nat. Rev. Nephrol..

[4] Omer Omer D., Pleniceanu O., Gnatek Y., Namestnikov M., Cohen-Zontag O., Goldberg S., Friedman Y.E., Friedman N., Mandelboim M., Vitner E. (2021). Human Kidney Spheroids and Monolayers Provide Insights into SARS-CoV-2 Renal Interactions. J. Am. Soc. Nephrol..

[5] Huang C., Wang Y., Li X., Ren L., Zhao J., Hu Y., Zhang L., Fan G., Xu J., Gu X. (2020). Clinical features of patients infected with 2019 novel coronavirus in Wuhan, China. Lancet.

[6] WHO Official Updates—Coronavirus Disease 2019. https://www.who.int/emergencies/diseases/novel-coronavirus-2019.

[7] Kang S.H., Kim S.W., Kim A.Y., Cho K.H., Park J.W., Do J.Y. (2020). Association between Chronic Kidney Disease or Acute Kidney Injury and Clinical Outcomes in COVID-19 Patients. J. Korean Med. Sci..

[8] Pecly I.M.D., Azevedo R.B., Muxfeldt E.S., Botelho B.G., Albuquerque G.G., Diniz P.H.P., Silva R., Rodrigues C.I.S. (2021). COVID-19 and chronic kidney disease: A comprehensive review. J. Bras. Nefrol..

[9] García-Granja P.E., Veras C., Aparisi Á., Amat-Santos I.J., Catalá P., Marcos M., Cabezón G., Candela J., Gil J.F., Uribarri A. (2021). Atrial fibrillation in patients with SARS-CoV-2 infection. Med. Clin..

[10] Russo V., Di Maio M., Mottola F.F., Pagnano G., Attena E., Verde N., Di Micco P., Silverio A., Scudiero F., Nunziata L. (2020). Clinical characteristics and prognosis of hospitalized COVID-19 patients with incident sustained tachyarrhythmias: A multicenter observational study. Eur. J. Clin. Investig..

[11] Levey A.S., Stevens L.A., Schmid C.H., Zhang Y.L., Castro AF 3rd Feldman H.I., Kusek J.W., Eggers P., Van Lente F., Greene T., Coresh J. (2009). CKD-EPI (Chronic Kidney Disease Epidemiology Collaboration). A new equation to estimate glomerular filtration rate. Ann. Intern. Med..

[12] Kellum J.A., Lameire N., Aspelin P., Barsoum R.S., Burdmann E.A., Goldstein S.L., Herzog C.A., Joannidis M., Kribben A., Levey A.S. (2012). Kidney Disease: Improving Global Outcomes (KDIGO) Acute Kidney Injury Work Group KDIGO clinical practice guideline for acute kidney injury. Kidney Int. Suppl..

[13] Dorjee K., Kim H., Bonomo E., Dolma R. (2020). Prevalence and predictors of death and severe disease in patients hospitalized due to COVID-19: A comprehensive systematic review and meta-analysis of 77 studies and 38,000 patients. PLoS ONE.

[14] Shahid Z., Kalayanamitra R., McClafferty B., Kepko D., Ramgobin D., Patel R., Aggarwal C.S., Vunnam R., Sahu N., Bhatt D. (2020). COVID-19 and Older Adults: What We Know. J. Am. Geriatr. Soc..

[15] Iaccarino G., Grassi G., Borghi C., Ferri C., Salvetti M., Volpe M. (2020). SARS-RAS Investigators. Age and Multimorbidity Predict Death Among COVID-19 Patients: Results of the SARS-RAS Study of the Italian Society of Hypertension. Hypertension.

[16] Gok M., Cetinkaya H., Kandemir T., Karahan E., Tuncer İ.B., Bukrek C., Sahin G. (2021). Chronic kidney disease predicts poor outcomes of COVID-19 patients. Int. Urol. Nephrol..

[17] Russo A.G., Decarli A., Valsecchi M.G. (2021). Strategy to identify priority groups for COVID-19 vaccination: A population based cohort study. Vaccine.

[18] Hansrivijit P., Qian C., Boonpheng B., Thongprayoon C., Vallabhajosyula S., Cheungpasitporn W., Ghahramani N. (2020). Incidence of acute kidney injury and its association with mortality in patients with COVID-19: A meta-analysis. J. Investig. Med..

[19] Thongprayoon C., Cheungpasitporn W., Hansrivijit P., Mao M.A., Medaura J., Bathini T., Chewcharat A., Erickson S.B. (2019). Admission Serum Potassium Levels in Hospitalized Patients and One-Year Mortality. Medicines.

[20] Kohsaka S., Okami S., Kanda E., Kashihara N., Yajima T. (2021). Cardiovascular and Renal Outcomes Associated With Hyperkalemia in Chronic Kidney Disease: A Hospital-Based Cohort Study. Mayo Clin. Proc. Innov. Qual. Outcomes.

[21] Hougen I., Leon S.J., Whitlock R., Rigatto C., Komenda P., Bohm C., Tangri N. (2021). Hyperkalemia and its Association With Mortality, Cardiovascular Events, Hospitalizations, and Intensive Care Unit Admissions in a Population-Based Retrospective Cohort. Kidney Int. Rep..

[22] Genovesi S., Vincenti A., Rossi E., Pogliani D., Acquistapace I., Stella A., Valsecchi M.G. (2008). Atrial fibrillation and morbidity and mortality in a cohort of long-term hemodialysis patients. Am. J. Kidney Dis..

[23] Baber U., Howard V.J., Halperin J.L., Soliman E.Z., Zhang X., McClellan W., Warnock D.G., Muntner P. (2011). Association of chronic kidney disease with atrial fibrillation among adults in the United States: REasons for Geographic and Racial Differences in Stroke (REGARDS) Study. Circ. Arrhythm. Electrophysiol..

[24] Voroneanu L., Ortiz A., Nistor I., Covic A. (2016). Atrial fibrillation in chronic kidney disease. Eur. J. Intern. Med..

[25] Boriani G., Fauchier L., Aguinaga L., Beattie J.M., Blomstrom Lundqvist C., Cohen A., Dan G.A., Genovesi S., Israel C., Joung B. (2019). European Heart Rhythm Association (EHRA) consensus document on management of arrhythmias and cardiac electronic devices in the critically ill and post-surgery patient, endorsed by Heart Rhythm Society (HRS), Asia Pacific Heart Rhythm Society (APHRS), Cardiac Arrhythmia Society of Southern Africa (CASSA), and Latin American Heart Rhythm Society (LAHRS). Europace.

[26] Schwartz A., Brotfain E., Koyfman L., Klein M. (2015). Cardiac Arrhythmias in a Septic ICU Population: A Review. J. Crit. Care Med..

[27] Paris S., Inciardi R.M., Lombardi C.M., Tomasoni D., Ameri P., Carubelli V., Agostoni P., Canale C., Carugo S., Danzi G. (2021). Implications of atrial fibrillation on the clinical course and outcomes of hospitalized COVID-19 patients: Results of the Cardio-COVID-Italy multicentre study. Europace.

[28] Richardson S., Hirsch J.S., Narasimhan M., Crawford J.M., McGinn T., Davidson K.W., Barnaby D.P., Becker L.B., Chelico J.D., the Northwell COVID-19 Research Consortium (2020). Presenting Characteristics, Comorbidities, and Outcomes Among 5700 Patients Hospitalized with COVID-19 in the New York City Area. JAMA.

[29] Moledina D.G., Simonov M., Yamamoto Y., Alausa J., Arora T., Biswas A., Cantley L.G., Ghazi L., Greenberg J.H., Hinchcliff M. (2021). The Association of COVID-19 with Acute Kidney Injury Independent of Severity of Illness: A Multicenter Cohort Study. Am. J. Kidney Dis..

[30] Hirsch J.S., Ng J.H., Ross D.W., Sharma P., Shah H.H., Barnett R.L., Hazzan A.D., Fishbane S., Jhaveri K.D., the Northwell COVID-19 Research Consortium (2020). Acute kidney injury in patients hospitalized with COVID-19. Kidney Int..

[31] Tarragón B., Valdenebro M., Serrano M.L., Maroto A., Llópez-Carratalá M.R., Ramos A., Rubio E., Huerta A., Marques M., Portolés J. (2021). Acute kidney failure in patients admitted due to COVID-19. Nefrologia.

[32] Walkey A.J., Wiener R.S., Ghobrial J.M., Curtis L.H., Benjamin E.J. (2011). Incident stroke and mortality associated with new-onset atrial fibrillation in patients hospitalized with severe sepsis. JAMA.

[33] Barbieri L.R., Sobral M.L., Gerônimo G.M., Santos G.G., Sbaraíni E., Dorfman F.K., Stolf N.A. (2013). Incidence of stroke and acute renal failure in patients of postoperative atrial fibrillation after myocardial revascularization. Rev. Bras. Cir. Cardiovasc..

[34] Ng R.R.G., Tan G.H.J., Liu W., Ti L.K., Chew S.T.H. (2016). The Association of Acute Kidney Injury and Atrial Fibrillation after Cardiac Surgery in an Asian Prospective Cohort Study. Medicine.

